# Case Series of Dengue Fever in Peripartum Period: Maternal and Foetal Outcome

**DOI:** 10.3390/idr12030013

**Published:** 2020-11-02

**Authors:** Yudianto Budi Saroyo, Ali Sungkar, Rima Irwinda, Raymond Surya

**Affiliations:** Department of Obstetrics and Gynecology Dr. Cipto Mangunkusumo Hospital, Faculty of Medicine Universitas Indonesia, Diponegoro No. 71, Central Jakarta 10430, Indonesia; yudibs@gmail.com (Y.B.S.); alisungkar@yahoo.com (A.S.); rima.irwinda@gmail.com (R.I.)

**Keywords:** dengue fever, pregnancy, maternal outcome, foetal outcome

## Abstract

*Introduction*: Dengue fever is a major public health problem in tropical and subtropical areas. There are not many studies concerning the complications of dengue fever in pregnancy. We present four serial cases of dengue fever in pregnancy. *Case illustration*: Three of four cases were delivered by caesarean section; two of them died during post-caesarean care. All cases had the lowest platelet level below 50,000/µL and were given platelet transfusion during and after delivery; they also showed abnormal liver function tests. For foetal outcome, none tested positive for dengue. *Discussion*: Complication of dengue infection depends on a combination of host and viral virulence. Regardless of prophylactic platelet transfusion, some studies revealed clinical bleeding in patients with dengue infection due to an intricate effect on the haemostatic system. The adverse foetal outcome may contribute because of placental circulation caused by endothelial damage with increased vascular permeability leading to plasma leakage. There is no national guideline for dengue fever in pregnancy. *Conclusions*: The management of dengue fever in pregnancy at the tertiary hospital is still suboptimal. Dengue fever around peripartum presents a higher risk of morbidity and mortality for the mother and therefore needs a multidiscipline team approach.

## 1. Introduction

Dengue fever (DF) is a major public health problem in tropical and subtropical areas. An estimated 50 million dengue infections occur worldwide with 500,000 people with dengue haemorrhagic fever requiring hospitalization annually. In the South Asia region, there were 232,530 dengue cases reported with a case fatality rate of 0.79 (2031 deaths) in 2009 [1]. In Indonesia, the annual incidence was around 35 to 40 per 100,000 and the case fatality rate reached 0.73% [2]. Dengue is defined as an acute febrile illness with one or more following signs or symptoms: intense headache, retro-orbital pain, myalgia, arthralgia, skin rash, leukopenia, and haemorrhagic manifestations [3]. In general, the WHO revised dengue infection in 2009 into dengue without warning sign (D–W), with warning sign (D+W), and severe dengue fever (SDF) [4].

In actuality, dengue in pregnancy can expose pregnant women to the risk of more severe infection in pregnant women than the general adult population and mother-to-child transmission before and during delivery [5]. Several previous studies showed that dengue in pregnancy can increase the risk of maternal haemorrhage, preterm labour, oligohydramnios, foetal deaths, and vertical transmission leading to neonatal thrombocytopenia requiring platelet transfusion [6,7]. The clinical presentation can be confused with Hemolysis, Elevated Liver enzyme, and Low Platelet count (HELLP) syndrome; thus, serology helps in distinguishing these two conditions [8].

There have not been many studies concerning the complications of dengue fever in pregnancy. We encountered four cases of dengue at the national referral Dr. Cipto Mangunkusumo hospital in 2019 during the peripartum period, with patients presenting fever onset several days prior to labour. They had different maternal outcomes, challenging us in the management of tertiary hospitals in Indonesia.

## 2. Case Illustration

### 2.1. Case 1

**Figure 1:**
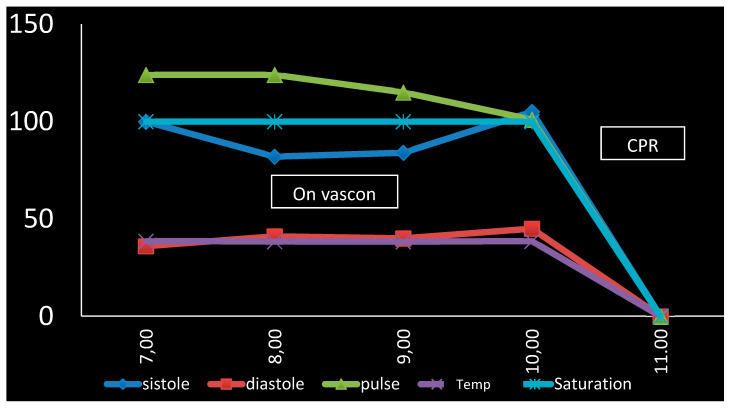
Vital chart of the last four hours.

**Figure 2:**
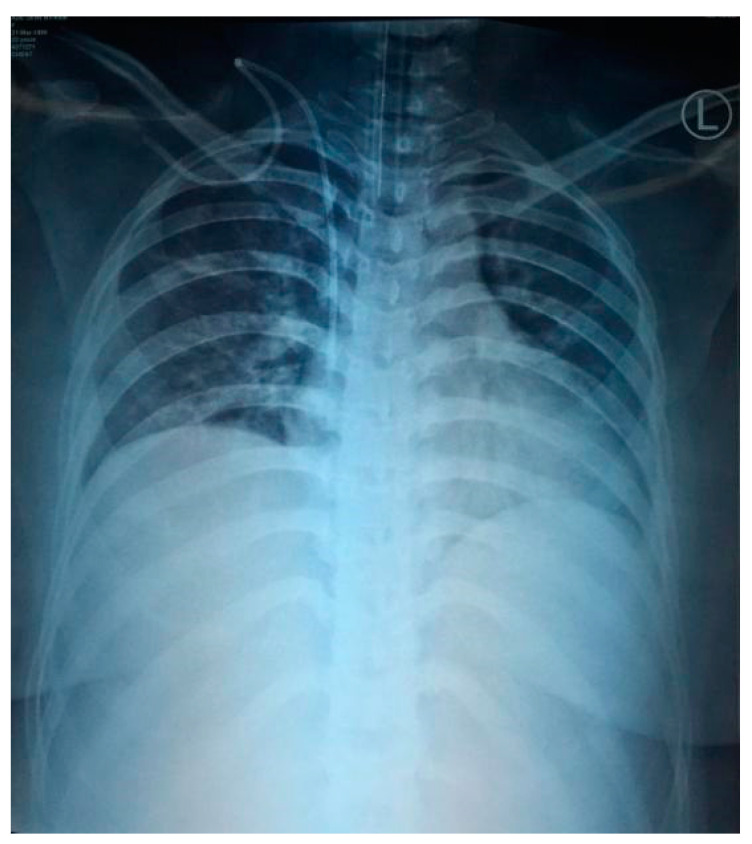
Thorax X-ray showed infiltrate on right suprahilar, perihilar, and paracardial with cranialization of broncho-vascular with differential diagnosis of pneumonia and lung oedema, suspected bilateral pleural effusion.

A 23-year-old woman was referred from the peripheral hospital in second-stage labour on G2P1 39 weeks of gestational age singleton live head presentation, previous c-section 1×, mother with SDF. She presented initially with high-grade fever, retro-orbital pain, nausea, and vomit for 5 days with 39 weeks of gestational age based on the last menstrual period (LMP). On physical examination, the blood pressure was 120/80 mmHg, heart rate 90 ×/min, temperature 37 °C. Blood examination revealed a normal haemoglobin level (13.9 g/dL), normal haematocrit level (41.3%), low platelet count (17,000/µL), and elevation of liver enzyme (AST 2923.5 U/L and ALT 1445.9 U/L), and she was also positive for Immunoglobulin M (IgM) and Immunoglobulin G (IgG) dengue serology with no result of NS1 antigen (Table 1). She was diagnosed as SDF due to elevated liver enzymes more than 1000 U/L. Her baby girl was born spontaneously, and her body weight was 2800 g with AS 7/8. The baby was tested for dengue transmission on day one, and the result was NS1, IgM, IgG and dengue serology from vein blood was negative. After delivery, the patient was sent to the ICU due to severe metabolic acidosis with respiratory failure threatening pH 7.17; partial pressure of carbon dioxide in arterial blood/pCO_2_ 22.7; partial pressure of oxygen in arterial blood/pO_2_ 135.9; bicarbonate/HCO3 8.4; O_2_ saturation 97.2%. She was immediately intubated and placed on mechanical ventilation Synchronized intermittent mandatory ventilation (SIMV) pressure support Positive End Expiratory Pressure (PEEP) 5, FiO_2_ 40% under midazolam sedation. Haemoglobin was decreased after vaginal delivery without any sign of active bleeding (Table 1). On the third day of ICU care (or day 8 of high-grade fever), the patient exhibited renal failure, signalled by increased urea and creatinine levels at two and three times the normal limit, respectively. She died on the fourth day of ICU care with the cause of death as multiple organ dysfunction due to SDF (Figure 1; Figure 2).

### 2.2. Case 2 

A 27-year-old woman was referred from peripheral hospital with G1 37–38 weeks of gestational age singleton live head presentation, mother with D+W, foetal with diaphragmatic hernia. She presented with 4 days of high-grade fever, headache, nausea, and vomit. On physical examination, blood pressure 110/80 mmHg, heart rate 87 ×/min, temperature 38 °C, and respiratory rate 18 ×/min. Blood examination revealed normal haemoglobin level (11.2 g/dL), normal hematocrite level (32.9%), low white blood count (3560/mm^3^) and platelet count (26,000/µL), slightly elevated liver enzyme (AST/ALT 47/86 U/L), positive IgM dengue serology and NS1 antigen, negative IgG dengue serology (Table 2). She was diagnosed as D+W due to comorbid condition of pregnancy and underwent caesarean section due to oligohydramnios, and the foetus had a congenital diaphragmatic hernia. A baby boy was born, and the body weight was 3210 g AS/5. The baby was sent to NICU due to diaphragmatic hernia, negative for NS1 antigen. After caesarean section, patient was treated in the ICU for three days without mechanical ventilation. Patient recovered and was discharged on day 9 of fever.

### 2.3. Case 3

A 31-year-old woman was referred from another hospital with G3P2 38 weeks of gestational age singleton live head presentation, previous c-section two times, mother with D+W. She presented with 6 days of high-grade fever without nausea and vomit. Physical examination revealed stable hemodynamic with a temperature of 37.5 °C. Blood examination showed normal haemoglobin level (13 g/dL), normal hematocrite level (38.7%), low white blood count (5230/mm^3^) and platelet count (47,000/µL), slightly elevated liver enzyme (AST/ALT 188.1/108.8 U/L), positive IgM and IgG dengue serology (Table 3). She was diagnosed as D+W and underwent caesarean section due to having two previous c-sections, not being in labour after elevated trend of platelet count on day 8 of fever. A baby boy was born, and the body weight was 2560 g with AS 8/10. The baby was roomed in with negative NS1 serology antigen. After caesarean section, patient was treated in the ward for two days and discharged on day 10 of fever.

### 2.4. Case 4

A 25-year-old woman was referred from another hospital with G2A1 36 weeks of gestational age singleton live head presentation, mother with D+W and preeclampsia with severe features. She presented initially with high-grade fever (39.8 °C) and retro-orbital pain for 2 days without either nausea or vomit. On physical examination, the blood pressure was 120/80 mmHg on anti-hypertensive agent, heart rate 83 ×/min, respiratory rate 20 ×/min, temperature 37 °C. Blood examination revealed normal haemoglobin level (12.2 g/dL), normal hematocrite level (35%), low platelet count (20,000/µL), elevated liver enzyme (AST 501 U/L and ALT 248 U/L), positive IgM, IgG dengue serology and NS1 antigen (Table 4). Patient received fluid and symptomatic therapy for the dengue and evaluation of foetal well-being through cardiotocograph. One day after admission (day 4 of fever), the cardiotocography showed suspicion two times and assessed as non-reassuring foetal status; therefore, and a caesarean section was performed. A baby girl was born, body weight was 2360 g with AS 6/8. The baby was tested for dengue transmission, and the result was that NS1, IgM, IgG dengue serology were negative. Patient was sent to ICU with mechanical ventilation of SIMV pressure support PEEP 5, FiO_2_ 40% under midazolam sedation. Around 8 h after cesarean section (day 5 of fever), there was active vaginal bleeding around 200 mL and recurrent vaginal bleeding around 300 mL in 15 h. Patient died on day 5 of fever due to disseminated intravascular coagulation (DIC) in D+W complication. 

Table 5 summarizes the characteristics of these serial cases.

## 3. Discussion

Dengue infection presents with a febrile period from 2 to 7 days followed by 3 to 4 days of defervescence phase leading to shock due to massive plasma leakage [9]. It is difficult to make a clinical assessment, diagnosis, treatment, and monitoring of dengue infection during pregnancy. There are various physiological changes in pregnancy with respect to the cardiovascular, respiratory, and hematologic system. At the end of the third trimester, plasma volume increases approximately 40% resulting in dilutional anaemia which masks the haemoconcentration which commonly occurs during the defervescence phase of D+W [10]. In our cases, the patient was referred to our hospital after 4 to 5 days of fever. Case number 1 and 4 still presented normal haematocrit level; however, they showed the two highest haematocrit levels in our case series ending in maternal death. This condition is in accordance with the theory of mimicking haemoconcentration in D+W for case 1 and SDF. In a case report by Hariyanto H, et al. [11] in Indonesia, the patient was in critical phase condition with normal haematocrit level; it was a state of shock with altered mental status and reduced perfusion.

The complication of dengue infection depends on a combination of host and viral virulence. The dengue virus replicates intracellularly and triggers an antigen–antibody complex causing immune-mediated cell destruction and production of cytokines and antibodies [12,13]. All cases presented with a positive IgM serology test and two cases with positive NS1 antigen. Patients with positive IgG and IgM serology test increase risk for exaggerated cytokine cascade response due to secondary dengue infection. In our cases, all patients clinically showed severe thrombocytopenia (<50,000/µL) with elevated liver enzymes. In another study, exaggerated cytokine cascade imposed a high risk for massive plasma leakage leading to the development of acute pulmonary oedema and ascites [13].

Until now, the time and mode of delivery have still been debatable. Not many countries have published their regulation. A national guideline from Sri Lanka in 2019 published that dengue fever in pregnancy should be managed by a multidisciplinary team. Induction or elective caesarean section is avoided during the critical phase of illness; if it cannot be delayed, the platelet should be increased over 50,000/µL. If there is indication or if it is mandatory, delivery should be conducted in the early febrile phase before the onset of the critical phase with platelet count above 130,000/µL. Indication and reason for delivery should be well documented. During the critical phase, vaginal delivery or caesarean section should be undertaken if the mother’s life is at risk or the patient develops spontaneous labour. Upon foetal distress, no intervention should be recommended in the critical phase [14]. Meanwhile, the American College of Obstetrics and Gynaecology (ACOG) recommends platelet transfusion to increase maternal platelet more than 50,000/µL before major surgery; meanwhile, epidural and spinal anaesthesia is considered acceptable with a platelet count of 70,000/µL to lower the risk of epidural hematoma [15]. Meanwhile, Brith Committee in haematology recommends prophylactic platelet transfusion for platelet level below 10,000/µL in the absence of bleeding manifestation, or in the case of massive systemic bleeding, platelet transfusion should be given in addition to red cell transfusion [16]. Case number 1 showed that the mother came with second-stage labour which could not be delayed; meanwhile, the platelet was 17,000/µL, so she received platelet transfusion. Case number 2 had CS performed due to foetal indication on the febrile phase with platelet level below 50,000/µL. She received a transfusion of platelets after CS. Meanwhile, case number 3 had CS performed after 8 days of fever and the dengue phase was recovered, in which there was an increasing trend of platelet level. Case number 4 was conducted CS due to foetal distress in the critical phase of dengue. Based on the recommendation above, case number 2 and 4 should be delayed after passing the critical phase or platelet level above 50,000/µL.

Regardless of prophylactic platelet transfusion, some studies revealed clinical bleeding in patients with dengue infection due to an intricate effect on the haemostatic system. Other risk factors coming from obstetric background should be considered such as uterine overdistention which might be manifested as uterine atony [17]. Hung LP et al. [18], in their case report, revealed postpartum haemorrhage in dengue patients due to polyhydramnios. Treatment with uterotonic drugs is essential for myometrial contraction and acts as a major factor preventing blood loss after natural delivery; however, there are abnormalities in the haemostatic system [17]. In our cases, there were three cases of bleeding manifestation after caesarean delivery without obstetric risk factors. Platelet count less than 30,000/µL was seen in dengue shock syndrome with increased risk of bleeding manifestations. The incidence of postpartum haemorrhage was reported between 10% and 19% in two studies [8,19]. Of three cases, haemoglobin level was dropped from 13.9 to 4.9 g/dL; 11.9 to 4.9 g/d; 14 to 6.8 g/dL, respectively. Thrombocytopenia is universally observed in dengue haemorrhagic fever; however, it is a poor indicator of bleeding manifestation. Coagulopathy describes the low fibrinogen level and prolonged aPTT which normally is increased due to a state of hyper coagulopathy in pregnancy. In all cases of manifestation with bleeding, low fibrinogen and prolonged aPTT were seen [20]. 

Adverse foetal outcomes may contribute because of placental circulation caused by endothelial damage with increased vascular permeability leading to plasma leakage [7,21]. A study from Tan et al. [21] showed that the vertical transmission rate of dengue is 1.6%. Another study by Basurko C et al. [19] showed a higher rate of vertical transmission up to 5.6%. In our serial cases, no vertical transmission was found, similar to an Indian study in eight pregnancies [22]. It is possible that the vertical transmission rate might be dependent on the severity of maternal dengue [23].

Based on the case series above, we suggest a national guideline for dengue fever in pregnancy regarding high prevalence of dengue fever in tropical countries, especially Indonesia. Apart from that, we recommended the recruiting of larger sample size populations to determine the maternal and foetal outcome of dengue fever in pregnancy. 

## 4. Conclusions

The management of dengue fever in pregnancy at the tertiary hospital is still suboptimal due to the lack of an available national guideline. Dengue fever around peripartum has a higher risk of morbidity and mortality for the mother; therefore, it needs a multidiscipline team approach.

## Figures and Tables

**Table 1:** Daily laboratory trend and therapy.

		Outside	Day 1	Day 2	Day 3	Day 4	Day 5	Day 6	Normal Value
Blood count	Haemoglobin (g/dL)	13.8	13.9	8.6	4.9	8	9.9	11.1	12–15
	Hematocrit (%)	41	41.3	24.7	14.9	22.8	28.3	31.9	36–46
	White blood cell (/mm^3^)	6600	18,110	23,300	41,200	35,720	26,270	33,030	5000–10,000
	Platelet (/µL)	27,000	17,000	22,000	28,000	38,000	103,000	116,000	150,000–400,000
Liver function	AST (U/L)		2923.5					5811	0–26
	ALT (U//L)		1445.9					3984	0–33
Coagulation profile	Prothrombin time (PT)		9.3 (10.6)			26.3 (10.3)	22.3 (10.4)	20.6 (10.4)	
	Activated partial thromboplastin time (aPTT)		44.9 (34.1)			82.2 (31.1)	54.9 (32.4)	40.3 (32.3)	
	d-Dimer (µg/mL)		0.2				0.6		0–0.3
	Fibrinogen (mg/dL)		225				146.8		150–400
Therapy given response to blood test	Packed red cell (mL)		None		232	307			
	Thrombocyte concentrate (mL)		321		327	None			
	Fresh frozen plasma (mL)		None		None	198			

**Table 2:** Laboratory trend and therapy.

		Day 1 (Outside)	Day 2 (Outside)	Day 3 (Outside)	Day 1	Day 2	Day 2	Day 4	Day 5	Day 6	Normal Value
Blood count	Hemoglobin (g/dL)	9.7	9.7	10.1	11.2	11.9	8.1	4.9	7.2	8.8	12–15
	Hematocrit (%)	29	29	31	32.9	34.5	24.8	15.2	21.5	27	36–46
	White blood cell (/mm^3^)	7180	5360	4000	3560	3960	11,770	17,060	25,520	21,170	5000–10,000
	Platelet (/µL)	160,000	125,000	85,000	26,000	15,000	37,000	80,000	141,000	217,000	150,000–400,000
Liver function	AST (U/L)				47		46			44	0–26
	ALT (U//L)				86		106			39	0–33
Coagulation profile	Prothrombin time (PT)				9.3 (10.7)		9.3 (10.7)	9.3 (10.5)			
	Activated partial thromboplastin time (aPTT)				56.7 (35.3)		47.2 (35.3)	49.4 (34.4)			
	d-Dimer (µg/mL)						0.3	1880			0–0.3
	Fibrinogen (mg/dL)						145.5	284.1			150–400
Therapy given response to blood test	Packed red cell (mL)					None	200	490	295		
	Thrombocyte concentrate (mL)					260	None	None	None		
	Fresh frozen plasma (mL)					None	235	None	None		

**Table 3:** Laboratory trend.

		Day 1 (Outside)	Day 2 (Outside)	Day 1	Day 2	Day 2	Day 3 (after CS)	Normal Value
Blood count	Hemoglobin (g/dL)	11.8	12.1	13	12.7	12.6	12.2	12–15
	Hematocrit (%)	36	37	38.7	38.7	37.7	36	36–46
	White blood cell (/mm^3^)	3510	4080	5230	7130	8310	10,300	5000–10,000
	Platelet (/µL)	146,000	95,000	47,000	74,000	141,000	224,000	150,000–400,000

**Table 4:** Daily laboratory trend and therapy.

		Day 1	Day 2	Day 3 (Post CS)	Day 3	Day 3 (after Bleeding)	Normal Value
Blood count	Hemoglobin (g/dL)	12.2	12.1	11	14	6.8	12–15
	Hematocrit (%)	35	36.2	32.4	42.3	21.3	36–46
	White blood cell (/mm^3^)	6270	7130	19,180	16,480	20,240	5000–10,000
	Platelet (/µL)	20,000	5000	6000	241,000	159,000	150,000–400,000
Liver function	AST (U/L)	501		416			0–26
	ALT (U//L)	248		205			0–33
Coagulation profile	Prothrombin time (PT)	9.9 (12.1)		10.1 (11.4)		23.8 (11.3)	
	Activated partial thromboplastin time (aPTT)	52.7 (36)		45 (33.9)		>180 (35.3)	
	d-Dimer (µg/mL)	1270				2410	0–0.3
	Fibrinogen (mg/dL)	153.5				95.9	150–400
Therapy given response to blood test	Packed red cell (mL)		None	206		306	
	Thrombocyte concentrate (mL)		300	505		None	
	Fresh frozen plasma (mL)		None	158		152	

**Table 5:** Summary of case series.

Patient	Age (Years)	Gestational Age (Weeks)	NS1 Antigen	IgM/IgG Dengue	Haematocrit (%) (Highest)	Platelet Count (10^3^/µL) (Lowest)	AST/ALT (U/L) (Highest)	Presenting Complaints	Mode of Delivery	ICU Admission	Platelet Transfusion	Maternal Outcome	Fetal Outcome
1	23	39	N/A	+/+	41.3	17	5811/3984	ROP, N, V	VD	Y	Y	Death	Normal
2	27	37–38	+	+/-	34.5	15	47/106	H, N, V	CS	Y	Y	Normal	Normal
3	31	38	N/A	+/+	38.7	42	188.1/108.8	N/A	CS	N	N	Normal	Normal
5	25	38	+	+/+	42.3	5	501/248	ROP	CS	Y	Y	Death	Normal

Note: N/A: not available; ROP: retroorbital pain; N: nausea: V: vomit; VD: vaginal delivery; CS: caesarean section; Y: yes; N: no.
